# Human sign-tracking and its relationship with attentional biases for alcohol

**DOI:** 10.1371/journal.pone.0358192

**Published:** 2026-09-28

**Authors:** Michelle Heck, Damien Lesenfants, Vincent Didone, Patrick Anselme, Etienne Quertemont

**Affiliations:** 1 Psychology & Neuroscience of Cognition - PsyNCog, Quantitative Psychology, Université de Liège, Liège, Belgium; 2 Department of Biopsychology, Ruhr-University Bochum, Bochum, Germany; Ritsumeikan Asia Pacific University: Ritsumeikan Asia Taiheiyo Daigaku, JAPAN

## Abstract

**Background:**

Pavlovian sign- and goal-tracking have been proposed as a translational framework for understanding individual vulnerability to addiction. In humans, eye‑movement–based Pavlovian Conditioned Approach (PCA) tasks and alcohol‑related attentional bias paradigms may capture complementary aspects of cue‑driven behavior, yet their relationship remains underexplored.

**Objectives:**

This study aimed (1) to test the hypothesized positive association between sign-tracking tendencies and alcohol-related attentional bias, and (2) further evaluate a computerized PCA paradigm for identifying human sign‑ versus goal‑tracking tendencies by attempting to replicate prior evidence linking sign-tracking to higher alcohol use.

**Methods:**

The main study included 159 healthy volunteers. Eye‑movement indices of sign‑ and goal‑oriented attention were collected, and latent profile analysis was used to derive PCA behavioral profiles. Alcohol‑related attentional bias was assessed using a free‑viewing task, while self‑report questionnaires measured alcohol consumption and personality traits. In an additional side experiment (n = 138), a gaze‑contingent, interactable lever was implemented as the conditioned stimulus (CS) to more closely approximate animal PCA procedures. Due to inconclusive outcomes, results from this experiment are reported in the supporting information.

**Results:**

Sign-trackers showed a modest tendency toward greater alcohol-related attentional bias than intermediate participants, although not compared with goal-trackers. No robust evidence emerged for differences in alcohol consumption across PCA profiles.

**Conclusions:**

The findings provide preliminary support for a positive association between sign-tracking tendencies and alcohol-related attentional bias, consistent with theoretical predictions, although the effect was small and should be interpreted cautiously. In contrast to previous reports, sign-tracking was unrelated to alcohol consumption. The attenuated effects likely reflect methodological constraints, including low reward salience and the passive nature of the task (e.g., fictitious token reinforcement). Further refinement of human PCA paradigms (particularly regarding conditioned stimulus properties, reward type, and participant engagement) is needed to clarify the boundary conditions of sign- and goal-tracking and strengthen their translational validity.

## Introduction

In everyday life, we are constantly exposed to cues that predict rewards, such as the sound of a coffee machine signaling an upcoming cup of coffee, the use of a lighter before smoking a cigarette, or the sound of a social media notification suggesting potential “likes” on a recent post. Through Pavlovian learning, these cues acquire incentive salience, enabling them to trigger appetitive behaviors such as reward approach, seeking, and craving [1–4].

Animal research has shown individual differences in how these cues are motivating and impact behavior. These differences have extensively been studied in animals using the autoshaping procedure, also known as Pavlovian Conditioned Approach (PCA). In PCA, a discrete conditioned stimulus (CS) is presented for a few seconds and is then consistently followed by the delivery of a food reward. During the CS presentation, individuals with a sign-tracking profile (STs) tend to approach and interact with the CS (e.g., a lever in rodent studies), whereas those with a goal-tracking profile (GTs) preferentially approach and inspect the location where the reward is to be delivered (e.g., the food tray). A third, intermediate profile (INTs), showing features of both behaviors, is occasionally reported [5]. Sign-tracking behavior is typically quantified using a response bias score, a numerical index based on the frequency or timing of contacts with the CS and the food dish location.

The sign- and goal-tracker (ST/GT) distinction is thought to reflect differences in incentive salience attribution: STs assign both a predictive and motivational value to the CS, making it highly attractive, whereas GTs attribute only a predictive value [5–7]. Although these phenotypes are relatively stable in laboratory animals, with evidence pointing to a genetic basis [e.g., 8, 9], they are not immutable. Decades of research indicate that several factors modulate their expression, including the type of CS (e.g., lever vs. tone; [10]), CS consistency [11], and early-life social experiences [12].

Animal studies further suggest that the ST/GT profiles have significant implications for addiction vulnerability [5,6]. STs acquire drug-taking behaviors (e.g., self-administration) more rapidly and exhibit greater susceptibility to cue-induced craving and relapse compared to GTs [5,6,9,13–18]. STs also show alterations in cognitive control, including reduced inhibitory control and increased impulsivity [19–21], extending the relevance of this phenotype to psychiatric vulnerability more broadly.

To establish the clinical utility of this model, robust evidence of its relevance in humans is essential. Over the past decade, numerous studies have adapted the ST/GT framework for human research, employing diverse materials, methods, and analytical strategies (for reviews see [22,23]). However, many paradigms deviate substantially from the original autoshaping procedure, complicating interpretation and limiting comparability. As a result, no standardized protocol currently exists for assessing sign- and goal-tracking in humans. Among the proposed approaches, eye-tracking during PCA tasks appears particularly promising [24–30]. From a translational perspective, this methodology (whether conducted in real-world settings using physical materials or by means of computerized tasks) offers the highest face validity, by preserving most crucial aspects of Pavlovian conditioning during autoshaping [23].

In a previous study, we developed a novel translational computerized task to assess individual differences in human STs and GTs, and examined their association with alcohol consumption [30]. Our findings indicated that individuals classified as STs showed overall higher alcohol use than GTs, a pattern observed using both the response bias score and latent profile analysis (LPA, data-driven) to characterize groups. It is important to note, however, that the response bias score distribution was strongly skewed toward goal‑tracking, reducing the robustness of the group contrasts. Moreover, we found no statistically significant relationship between impulsivity measures and sign‑tracking magnitude, although a small, non‑significant trend emerged between sign‑tracking and positive urgency (r = .25).

Overall, human studies support the relevance of ST/GT behavioral phenotypes as potential markers of vulnerability to substance use, in line with findings from animal research. Associations have been reported between sign‑tracking and drug or alcohol use [30–35], as well as with impulsivity‑related traits [25,26,29,34,36]. Because STs attribute incentive salience to conditioned stimuli associated with reward, they should also differ from GTs in their susceptibility to develop drug‑related attentional biases, defined as a disproportionate allocation of attention toward drug‑associated cues and theorized to reflect heightened attribution of incentive salience to drug‑predictive stimuli.

Alcohol attentional bias is well documented, and research indicates consistent associations with alcohol consumption and craving, particularly in subclinical populations ([37–41], although see [42]). However, the results are mixed and less consistent in individuals with severe alcohol use disorder [37,43,44]. Notably, alcohol attentional bias is dynamic and fluctuates with factors such as craving state [45], in vivo alcohol exposure [46], and immediate alcohol expectancies [47]. One intriguing hypothesis is that the heterogeneous findings observed in alcohol attentional bias research, especially in clinical samples, may reflect individual differences in ST/GT tendencies. Such differences could determine whether attentional bias plays a meaningful role in alcohol use disorder for some individuals but not others [37]. Although attentional bias and sign-tracking may represent distinct phenomena (particularly given the absence of a true animal equivalent for attentional bias studies), they both capture aspects of ‘automatic’ or “irrational” behavior, manifesting as either physical approach or the allocation of attentional resources toward reward-associated cues. Both have been linked to addiction-related processes, yet, to our knowledge, they have never been examined together to determine if they are significantly associated.

Building on the results of our initial study [30], the present research aimed to address this gap by examining whether ST, GT, and INT profiles are associated with alcohol‑related attentional bias. Specifically, we tested whether individuals displaying stronger sign‑tracking tendencies would also exhibit greater attentional bias toward alcohol‑related cues. We hypothesized a positive relationship between sign‑tracking tendencies and alcohol attentional bias. This study involved administering the computerized PCA task together with a free‑viewing alcohol attentional bias task.

As part of the broader project, we also conducted a variant of the experiment. In this variant, we tried to approximate the animal paradigm more closely with respect to the predictive stimulus (sign). The sign consisted of the image of a lever that participants could manipulate using their gaze (the goal stimulus was depicted as a simple rectangle). The lever moved horizontally in response to gaze direction, reflecting evidence from animal studies that manipulability of the CS is critical for eliciting sign‑tracking behavior [10,48], but see [49]. However, we suspected that this methodological modification could undermine the opportunity to validly capture sign‑ versus goal‑tracking behavior. Indeed, the interactive lever could potentially alter participants’ interpretations of the task: action-related stimuli such as levers are typically associated with contingent outcomes in human settings, potentially prompting an operant‑like strategy in which rewards were perceived as dependent on lever manipulation rather than on purely Pavlovian contingencies. Aside from the representation of the sign and goal stimuli, this experimental variant was identical to the present experiment. For the sake of transparency and conciseness, the methodological details, results, and discussion related to this separate experiment are reported in Appendix 1 (Supporting Information S1).

## Materials and methods

### Participants

The sample of this study included 159 volunteers (102 females, 56 males and 1 identified as non-binary; mean age = 21.70, sd = 2.72, see Table 1 for sample description). They completed an online screening survey to check for inclusion criteria, which were: no history of severe neurological diseases, no regular substance use (more than twice in the last two months, except for tobacco and alcohol), no colorblindness, no medicated attention deficit hyperactivity disorder. All participants had to be aged between 18 and 35 and be at least occasional alcohol consumers (abstinent people were not included). All participants had normal or corrected-to-normal vision.

**Table 1:** Sample Description of Experiment 1.

Variable	Median (std)	Min-Max
**Age**	21 (2.72)	18-33
**Education Level**	14 (1.81)	9-21
**AUDIT-C**	5 (2.46)	1-12
**Recent Drinking Index**	12 (70.60)	0-720

*Note.* Education Level = number of successful school years starting from elementary school; AUDIT-C = short version (consumption) of the Alcohol Use Disorder Identification Test, Recent drinking index = number of alcoholic drinks consumed during the past 2 weeks x number of drinking occasions during the past 2 weeks.

Based on the effect size of the previous study, a minimum of 138 participants were required to achieve .80 power (with an alpha of 0.5). However, more participants were recruited, to account for the non-parametric analyses and data loss due to quality check criteria (for eye-tracking data), as well as careless responding. Overall, 180 subjects took part in the whole study. 20 subjects were excluded from the analysis due to at least one of the data quality check criteria (see Measures – Eye-gaze). One participant was excluded after it was determined that he had previously participated in a related experiment conducted by our laboratory on the same topic.

### Ethics statement

The study was conducted in accordance with institutional guidelines and the Declaration of Helsinki. It was approved by the Ethics Committee of the Faculty of Psychology, Speech Therapy, and Educational Sciences of the University of Liège. Participants were recruited via word of mouth, social media or advertising posters. The study title was a dummy (related to intelligence and reaction time measures), to avoid disrupting behaviors that we wanted to study. After filling out the online screening, participants who met inclusion criteria were contacted to take part in the experimental phase of the study (on-site). All participants gave written informed consent to take part in the study. Recruitment and testing took place from October 1^st^ 2024 to December 19^th^ 2025. Importantly, unlike our previous experiment [30], participants in the present study did not receive compensation via a prize draw (i.e., a lottery-based incentive in which participants can win a reward) or any other method.

### Procedure

The testing session consisted of three phases: (1) pre-experimental questionnaires (2 minutes), (2) the battery of experimental tasks (approximately 25 min, this phase was interrupted by a break after the first two blocks of the PCA task, during which participants completed a questionnaire), and (3) post-experimental questionnaires (10 minutes). Participants were tested in a quiet room. Their head was restrained by a chinrest at a distance of 70 cm from the screen. They were allowed to take short breaks between the different steps to avoid fatigue. At the end of the experimental session, the real purpose of the study was revealed to the participants, after which they had the possibility to give a second written informed consent.

### Stimuli

Before starting the ST/GT task, participants were instructed that they could collect a certain number of points in the next tasks, and that more points collected would unlock more glorious expertise badges. We chose accumulation of points and unlocking of badges to reflect reward amount because this procedure does not impact the participants’ motivation to seek rewards [33,50]. The stimuli for the PCA task and the attentional bias task were as follows:

#### Goal and sign pictures.

The sign cue was a medium blue rectangle patterned with light blue dots, while goal location was represented by a medium blue rectangle patterned with light blue squares, following the design in [30]. The sign and goal pictures were located on middle top or middle bottom of the screen and counterbalanced for location across participants.

#### Reward stimuli.

Either a yellow 5-point coin or a yellow 10-point coin in the design of a casino coin.

#### Expertise badges.

Fancy and colorful badges representing performance (ranging from the categories bronze, silver, gold, platinum, diamond, and expert).

#### Test pictures (free-viewing attentional bias task).

Ten pairs of pictures (taken from the royalty-free picture stock “Pixabay”) of approximately the same color.

#### Stimuli pictures (free-viewing attentional bias task).

Sixty pairs of alcohol and neutral pictures (10 in each of the following categories: beer, wine, champagne, liquor, cocktails, environments). The alcohol pictures were selected from the Wake Forest Alcohol Imagery Set (WFAIS) [51], through consensus with experts and non-experts among colleagues (PIs and PhD candidates). The neutral pictures were created to resemble each alcohol picture while not being directly associated with alcohol, using either DALL-E (OpenIA), or by taking our own pictures. They were matched on color and brightness (using Adobe Photoshop, version 2024). All the pairs of pictures underwent a pilot validation phase, during which independent participants were asked to rate the alcohol-relatedness of each picture (on a visual analogue scale ranging from 0 to 10), to make sure that the neutral stimuli were considered alcohol-unrelated. Mean score ranges for each category are shown in Appendix 2 (Supporting Information S2). One neutral picture, scoring 3.8, was replaced before the experiment. An illustration of two pairs of pictures is shown in Fig 1.

**Fig 1:**
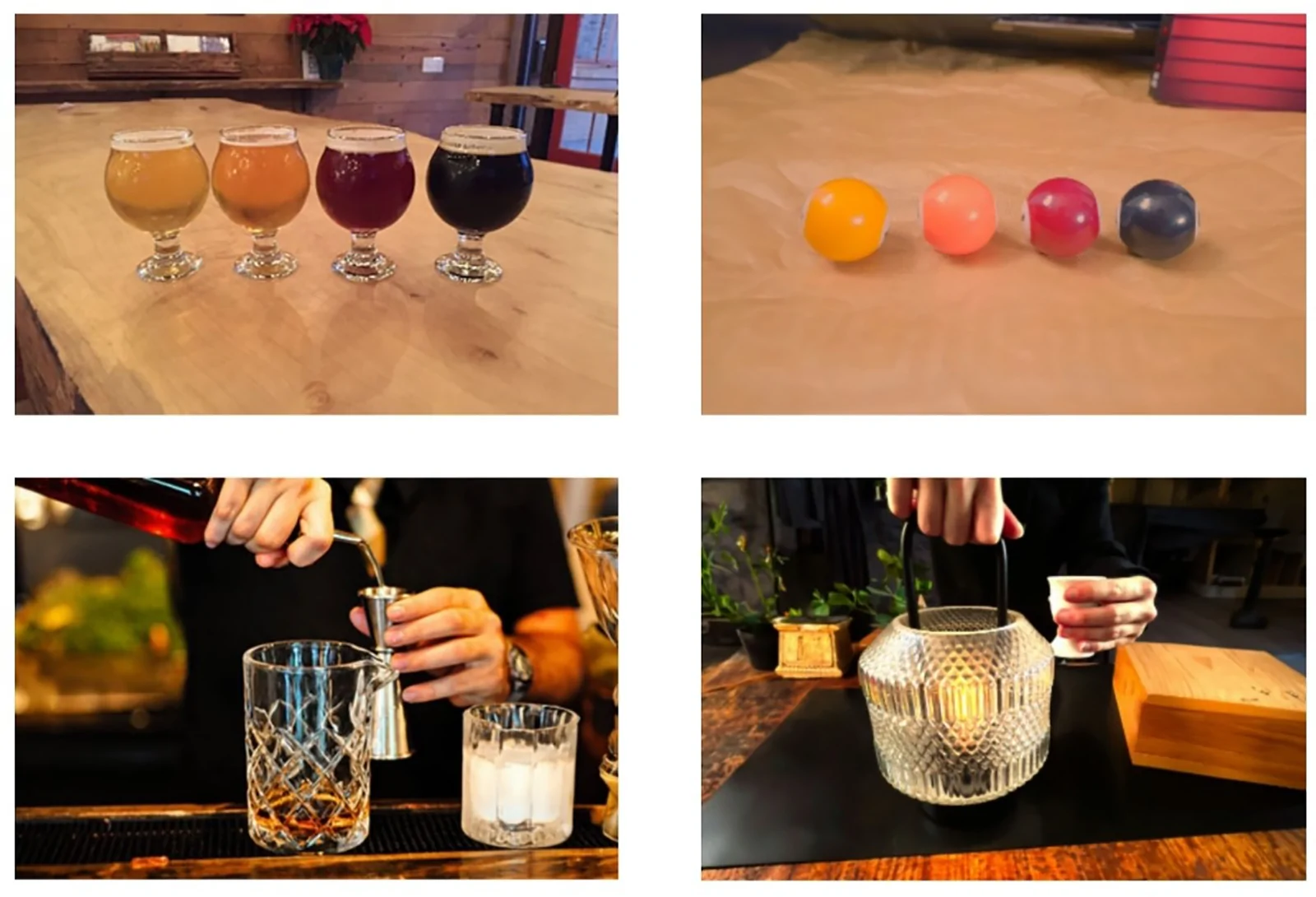
Examples of stimuli in the attention bias task. Examples of picture pairs, for beer (above) and liquor (bottom).

The stimuli were presented on a computer screen (1920 x 1080 pixels) with a grey background. A battery of three computer tasks was implemented: (1) the simple reward task, (2) the PCA task, and (3) the free-viewing attentional bias task. The three tasks were run on a homemade custom Matlab software (version R2019b).

### Simple reward task

This task involved a permanent rectangle (goal) on the screen, counterbalanced for location across participants (bottom or upper part of the screen). The coin appeared for 1.5 seconds on the goal shape, and the sound of a falling coin could immediately be heard when the participant pressed the key to collect the reward. There was a 20% chance that the coin was a “10-point coin” (instead of a 5-point coin) to maintain curiosity and avoid monotony during the task. The aim of this first task was for the participants to learn where to collect the rewards, and it was designed to mimic the magazine training phase in animal models, where rewards are regularly delivered without any CS presentation. The simple reward task consisted of 10 trials, with a variable inter-trial interval (ITI) from 4 to 8 seconds. Prior to starting the task, participants were instructed as follows: “*During this task, objects will be displayed in a blue rectangle. These will allow you to earn points. Your objective is to respond as quickly as possible to their presentation by pressing the “enter” key*.”

### PCA task

This task was developed in [30] and was designed to match the autoshaping procedure used in animal ST/GT studies. The goal shape was permanently present in the lower or upper part of the screen. In the opposite area of the screen, the sign cue (CS) appeared for 3 seconds before a coin was shown on the goal shape for 3 seconds. The CS and the coin reward were associated with an 80% chance to hold interest in the task.

During reward presentation, pushing the “enter” button produced the sound of a falling coin informing participants that the reward was being collected. The task consisted of 2 blocks of 20 trials with a variable inter-trial interval (ITI) ranging from 4 to 8 seconds. Like in the previous task, there was a 20% chance that a “10-point coin” was obtained.

Prior to starting the task, participants were instructed as follows: “*This task is similar to the first one, except that we’re no longer measuring your reaction time here. You can also score points. Use the “enter” key as before. Please keep your attention on the computer screen and don’t look at the keyboard.*”

### Free-viewing attentional bias task

The task was divided into two parts, a training phase and a test phase [52].

#### Training phase.

The training phase consisted of 10 trials, each presenting a pair of neutral images for 2500 ms on either side of a central fixation cross. The order of image pairs was randomized.

Each trial proceeded as follows: the image pair was first displayed for 2500 ms, followed by a central fixation cross presented for 1500 ms and then by a variable inter-trial interval (ITI) ranging from 2 to 4 seconds, during which a blank screen was shown. Participants received the following instructions: “*You will now see pairs of images. Please look at these images as you would at pop-ups on your computer. Between each pair, a fixation cross will appear briefly. Please focus on this cross before the next trial begins. You’ll start with 10 practice trials. Press the space bar to start.*”

#### Test phase.

The test phase followed the same procedure as the training phase, with identical trial structure and timing. Each trial consisted of the presentation of a pair of images for 2500 ms, followed by a fixation cross (1500 ms) and a variable inter-trial interval (0.5–1.5 s). In most trials (n = 60), an alcohol-related image was presented alongside a matched neutral image (left–right position counterbalanced). In the remaining trials (n = 12), both images were neutral, serving as control trials. The order of trials was randomized. The task comprised 72 trials in total and lasted approximately 8 minutes. Prior to starting, participants were instructed to perform the task in the same manner as during the training phase.

### Data collection

#### Questionnaires.

In the screening phase, all participants completed socio-demographic online questionnaires including the history of neurological problems that could affect cognitive functioning, as well as regular use of tranquilizers or illicit drugs. They also completed the short (consumption) version of the alcohol use disorder identification test (AUDIT-C) [53] and the short version of the French Impulsive Behavior Scale (UPPS-P) [54].

In the pre-experimental phase, all participants completed electronic versions of the following questionnaires. First, *The Edinburgh handedness inventory* [55], which assesses handedness through 22 items (the 11-item version was used). Second, *The Karolinska Sleepiness Scale* (KSS) [56], which measures the subjective level of fatigue at a particular time of day based on a nine-point Likert-type scale (both questionnaires were used, but not included in this report). Third, *The Reinforcement Sensitivity Theory of Personality Questionnaire* (RST-PQ [57],) which assesses six dimensions. In the present study, we only measured three dimensions (total number of items = 24): Reward interest, Goal-drive persistence, and reward reactivity. Finally, an attention check item was embedded in this questionnaire in order to identify and exclude careless responders after collecting the data [58]. The item read: “*Please select “completely agree” for this item”, on a 6-level scale from “completely disagree” to “completely agree”*.

In the post-experimental phase, participants completed electronic versions of the following questionnaires. First, *The Karolinska Sleepiness Scale* (KSS) [56]. Second, the short *Depression, Anxiety, Stress Scale* (DASS-21) [59], which yields three subscale scores (21 items). A total score can be computed, representing general psychological distress. Third, the French version [60] of the *Alcohol Use Disorders Identification Test* (AUDIT; [61]) consisting of 10 multiple-choice items measuring alcohol consumption, alcohol dependence, and alcohol-related problems. Higher scores reflect a high probability of harmful alcohol use. Further *Questions about current alcohol consumption*, consisting of the following two questions: “How many drinks have you had in the last 14 days?” and “On how many occasions have you drunk alcohol in the last 14 days?” were also added. A drinking occasion index was computed by multiplying these two numbers. We also measured the following item: “How many times have you engaged in binge drinking in the past month? (Defined as consuming >5 drinks within 2 hours for men and >4 drinks within 2 hours for women.). Finally, *The Brief Assessment Tool for Compulsivity Associated Problems* (BATCAP) [62], which assesses a range of compulsive symptom types. It covers alcohol use, gambling, binge eating and excessive Internet use (as well as contamination, checking, and ordering compulsions). In this study, we only used the addiction-related components. A “primary addiction score” was computed (score of the top-scoring domain across addiction behaviors).

#### Eye-tracking.

A Tobii 5 eye-tracking system (with an image sampling rate of 133 Hz) was mounted on a 60.5 cm diagonal computer screen. The eye tracker was calibrated using a six-point procedure at the beginning of the experiment. A chinrest was used to standardize the head-screen distance at 70 cm. Tobii Experience software was used to calibrate the eye-tracking system.

To compute eye gaze-based ST/GT scores, the screen was virtually separated into a 3-by-3 matrix (total of 9) regions of interest (ROI). Eye gaze position was collected throughout the PCA task. The two main ROIs used to compute the scores were the upper center and the bottom center of the screen, where the goal and the sign stimuli were located. The in-between region (middle center) was also of interest for LPA.

We used a custom implementation of the Velocity Threshold Algorithm for fixation identification. First, eye position in pixels was converted to visual degrees. Then velocity was computed in degrees/sec for axes X and Y. To distinguish fixations from saccades, we used individualized thresholds for each subject based on their gaze velocity during mandatory fixations in the training phase of the free-viewing task. Each threshold was computed by adding 1.95*standard deviation to their mean gaze velocity during fixations (= confidence interval of 95%). In probability and statistics, the 97.5th percentile point of the standard normal distribution is a number commonly used for statistical calculations. The approximate value of this number is 1.95, meaning that 95% of the area under a normal curve lies within approximately 1.95 standard deviations of the mean. Because of the central limit theorem, this number is used to approximate 95% confidence intervals.

Eye movements were considered fixations when they were strictly below the personalized threshold. Unphysiological too short fixations (<100 ms) were removed to smoothen eye-gaze data. For each identified fixation, duration and ROI were collected. Two outcome measures were obtained: Fixation count (number of fixations) and dwell time (total fixation time on each ROI), two classic parameters when working with fixation-based measures [63].

Three criteria were used for data quality check and poor quality data exclusions: A participant was excluded (1) if the gaze stagnated in the middle zone throughout the entire task (perhaps an indicator of poor calibration or other technical problem, n = 0), (2) if there were more than 30% of gazes at the outer edge of the screen (5% of the screen) compared to the rest of the screen (poor calibration, n = 0), and (3) if on average the individual gazed less than 1 second per CS presentation at either the ROI (goal and sign) or the center of the screen (because of too few data to compute a meaningful sign-tracking score, n = 20).

**Eye-tracking based sign- and goal-tracking scores.** Eye-tracking based ST/GT scores were computed as follows: dwell time on the “goal” was subtracted from dwell time on the “sign” and divided by the total dwell time on both ROIs (during the 3s CS presentation). Thus, the ST/GT score ranged from −1–1, with negative scores corresponding to “goal-tracking” phenotypes, positive scores representing “sign-tracking” phenotypes, and 0 meaning a rather “intermediate” tendency. This score was computed for all blocks of the PCA task, for the number of fixations as well as for the total dwell time. Since some time was required for the participants to become familiar with the task and for specific conditioned responses to emerge [25,26,29], only the second block score was considered in the main analyses. Groups of STs, INTs, and GTs were created via LPA.

**Attentional bias.** For the free-viewing attentional bias task, two bias scores were computed [52], each representing different stages of attentional processing. An automatic attention score, computed as the percentage of first gazes on the alcohol vs neutral pictures, and a sustained attention score, computed as the total percentage of time the gaze was recorded on the alcohol vs neutral picture.

## Statistical analyses

All analyses listed here were planned in the pre-registration (osf.io), and run with R 4.5.1 [64]. Basic data importation, processing, and plotting were done using *readxl* [65], *tidyverse* [66], and *ggpubr* [67]. ANOVAs and related procedures used *rstatix* [68], *car* [69], and *lmtest* [70]. Effect sizes were computed with *lsr* [71], correlations with *psych* [72], and latent profile analyses with *tidylpa* [73]. Alpha was set at 0.05, but corrections for multiple correlations were applied as indicated.

Given that the questionnaires were administered electronically, no missing data were expected, as participants were required to answer all questions in order to proceed. However, missing data occurred due to system bugs: one participant had missing data for the KSS and another for the RST-PQ. These participants were excluded from the relevant analyses, as these measures were not part of the primary analyses. There were also missing data for the attentional bias task due to an insufficient number of trials with gazes recorded in the ROIs (defined as at least one third of trials). Participants with missing AB data were excluded from the relevant analyses (N = 5).

The primary hypothesis examined whether sign-tracker profiles were associated with levels of alcohol consumption. To address this, we conducted a LPA to determine whether the previously established three-profile model (STs, GTs, and INTs) provided an adequate fit to the present data and could therefore be used to classify participants. Indicator variables for the LPA were derived from the second block of the sign-tracking task and initially included six attentional measures: fixation counts and total dwell time directed toward the goal cue, sign cue, and middle zone. Prior to conducting the LPA, correlations among indicators were examined. Because sign-directed fixation counts and dwell time were nearly perfectly correlated (*r* = .98), and the two middle-zone measures were also highly correlated (*r* = .95), these pairs of variables were standardized and averaged to create composite indices. This data-reduction step was intended to minimize redundancy among indicators that appeared to capture highly similar aspects of attentional engagement. In contrast, goal-directed fixation counts and dwell time showed only a weak association (*r* = .20) and were therefore retained as separate indicators, as they likely reflected distinct dimensions of attentional engagement with the goal stimulus. Table 2 shows the intercorrelations between LPA indicators. Overall, associations were small to moderate, with no evidence of problematic multicollinearity (all |*r*| < .80). Negative correlations between cue-directed and goal-directed indicators were expected, as the fixed duration of each trial creates an inherent trade-off in attentional allocation across ROIs.

**Table 2:** Intercorrelations among LPA indicators.

Variable	1	2	3	4
1. Sign attention	—			
2. Middle attention	0.29**	—		
3. Goal dwell time	−0.64**	−0.54**	—	
4. Goal fixation count	−0.005	−0.33**	0.21*	—

*Note.* **p < 0.001, *p < 0.01.

This indicator reduction strategy was not specified in the preregistration. However, it was implemented to improve the parsimony and interpretability of the latent profile model by reducing the influence of highly redundant indicators while preserving conceptually distinct information.

We estimated a series of models with increasing numbers of profiles. Model selection was guided by both theoretical considerations and multiple fit indices [74], including the Bayesian Information Criterion (BIC), Akaike Information Criterion (AIC), and entropy (values approaching 1 indicating better classification). Following some work [75], our model specification allowed covariances among indicator variables to be freely estimated within profiles, while variances and covariances were constrained to equality across profiles.

After establishing consistent profiles, we tested group differences in alcohol consumption severity (AUDIT-C scores, short version of the Alcohol Use Disorder Identification Test) using a Kruskal–Wallis rank-sum test (given that ANOVA assumptions were not met).

Secondary and Tertiary Hypotheses: We further examined whether ST/GT/INT profiles were associated with attentional bias for alcohol cues. Specifically:

- Automatic orienting: A Kruskal–Wallis test assessed differences in the proportion of initial fixations on alcohol-related versus neutral stimuli (calculated as the number of alcohol-first/total trials).
- Controlled attention: Another Kruskal–Wallis test evaluated differences in the percentage of total fixation time allocated to alcohol versus neutral images.

To validate the conceptual integrity of the alcohol attentional bias task, we conducted correlational analyses between alcohol consumption measures and the two attentional bias indices, expecting positive associations as theoretically predicted. To control for multiple comparisons, we applied the Benjamini–Hochberg correction [76].

Exploratory correlational analyses (Spearman’s rho), pre-registered prior to data collection, examined relationships between ST/GT scores and alcohol consumption variables, as well as personality-related constructs (e.g., impulsivity, reward reactivity, psychological distress). Benjamini–Hochberg correction was also applied [76].

Finally, in an exploratory (and non–pre-registered) analysis, we examined whether dwell time directed toward the sign and the goal varied across trials and experimental blocks for the three behavioral profiles identified via LPA. To do so, we fitted a linear mixed‑effects model that included fixed effects of profile, trial number, block, and their interactions (profile × trial and profile × block). The corresponding results are reported and illustrated in Appendix 3 (Supporting Information S3).

## Results

### Hypothesis 1. Are the “sign-tracker profiles” related to alcohol consumption levels?

The best model based on fit indices was the 2-profile model (AIC = 1484.97, BIC = 1543.27, entropy = 0.91). However to be consistent with previous results and a priori planned analyses, the 3-profile model was retained (AIC = 1417.00, BIC = 1490.66, entropy = 0.87).

Table 3 shows the fit indices for the three models. Posterior probabilities (i.e., probabilities for each participant to belong to each of the three profiles) indicated mediocre confidence in the most likely profile membership. For profile 1, the probabilities ranged from 0.57 to 1.00 (mean = 0.90, sd = 0.13). For profile 2, they ranged from 0.69 to 1.00 (mean = 0.97, sd = 0.08). For profile 3, they ranged from 0.50 to 0.99 (mean = 0.95, sd = 0.10). Fig 2 shows means and standard errors for the standardized indicator variables in the three profiles. Based on the examination of these graphs, groups 1, 2, and 3, were labeled respectively, “INT”, “ST”, and “GT”.

**Table 3:** Model Fit Indices for the Three Latent Profile Analyses in Experiment 1.

Tested Model	AIC	BIC	Entropy	Profile Sizes	Bootstrapped Likelihood Test (p-value)
**1 Profile**	1537.89	1580.85	–	–	–
**2 Profiles**	1484.97	1543.27	0.91	Profile 1: 24% Profile 2: 76%	.01
**3 Profiles**	1417.00	1490.66	0.87	Profile 1: 29% Profile 2: 14% Profile 3: 57%	.01

*Note*. AIC=Akaike information criterion; BIC = Bayesian information criterion.

**Fig 2:**
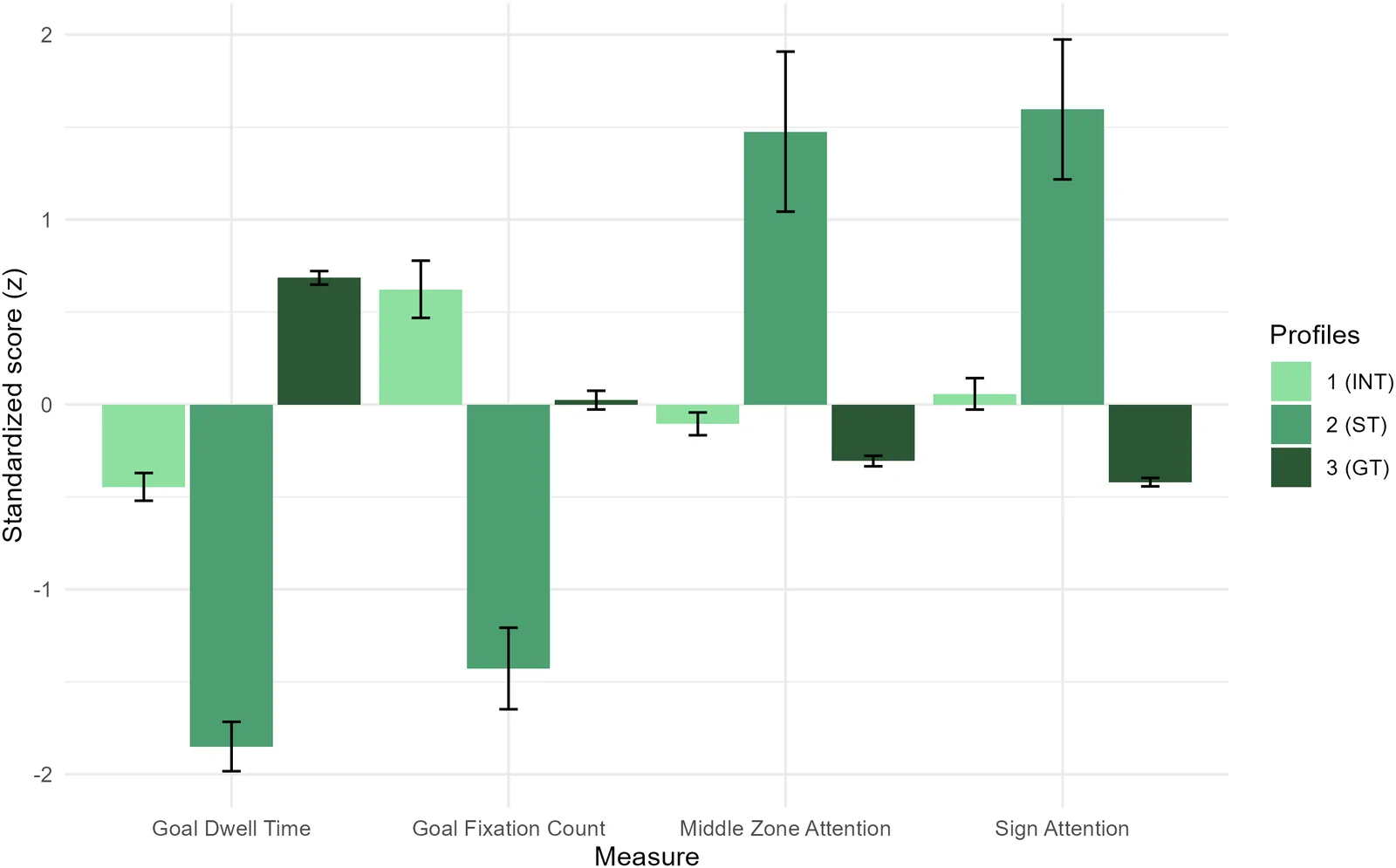
Standardized Eye-tracking based Indicator variables in the Three LPA-Extracted Profiles. The figure shows the means and standard errors. All pairwise differences are significant at at least p < 0.01.

The Kruskal-Wallis rank-sum test, assessing the differences in alcohol consumption (AUDIT-C) in these profiles, did not show a statistically significant effect, possibly due to the uneven distribution of participants across groups (χ² = 4.25, *p* = 0.11, η^2^(H) = 0.01, 95% CI = [−0.07, 0.04]). Fig 3 shows the distribution of AUDIT-C scores in STs, GTs, and INTs, based on LPA-created profiles.

**Fig 3:**
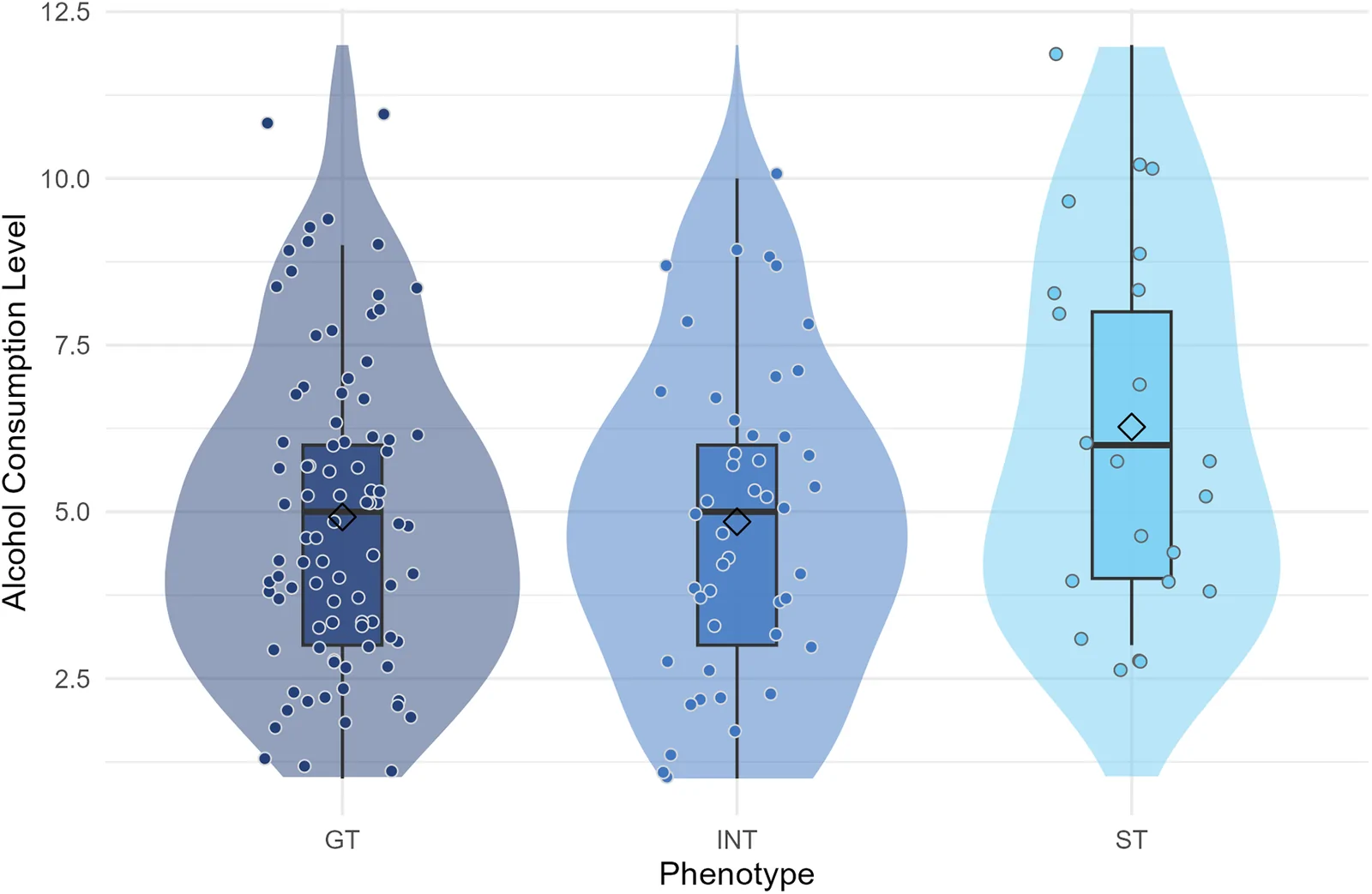
Alcohol Consumption Level in STs, GTs and INTs based on LPA. Horizontal lines are the medians, the lower and upper limits of the boxes are lower and upper quartiles, the ends of the whiskers represent the lower and upper extremes. The lines around the boxes represent the distribution of the data in each group.

### Hypothesis 2. Is There a Relationship Between Human Sign-Tracking and Alcohol Attentional Bias?

To test whether the ST/GT profiles differed in *automatic orienting* (operationalized as the proportion of trials in which participants initially fixated on the alcohol-related image), a Kruskal-Wallis test was conducted since ANOVA assumptions were not met. The analysis revealed a statistically significant group effect, (χ²(2) = 7.24, *p* = 0.02). However, the associated effect size was small (η²[H] = 0.03, 95% CI [−.05, .08]) and the confidence interval included largely 0, indicating that profile membership accounted for only a limited proportion of the variance in initial alcohol orienting.

Follow-up pairwise Wilcoxon tests with Benjamini–Hochberg correction showed that this effect was primarily driven by a difference between STs and INTs (*p* = .03). Comparisons between GTs and INTs (*p* = .12) and between STs and GTs (*p* = .10) did not reach statistical significance. Fig 4 illustrates the distribution of initial alcohol orienting across GT, INT, and ST profiles derived from the LPA. Although the overall group effect reached statistical significance, the unequal group sizes and the small effect size warrant caution in interpreting this finding, as statistical significance may overstate the practical importance of the observed differences.

**Fig 4:**
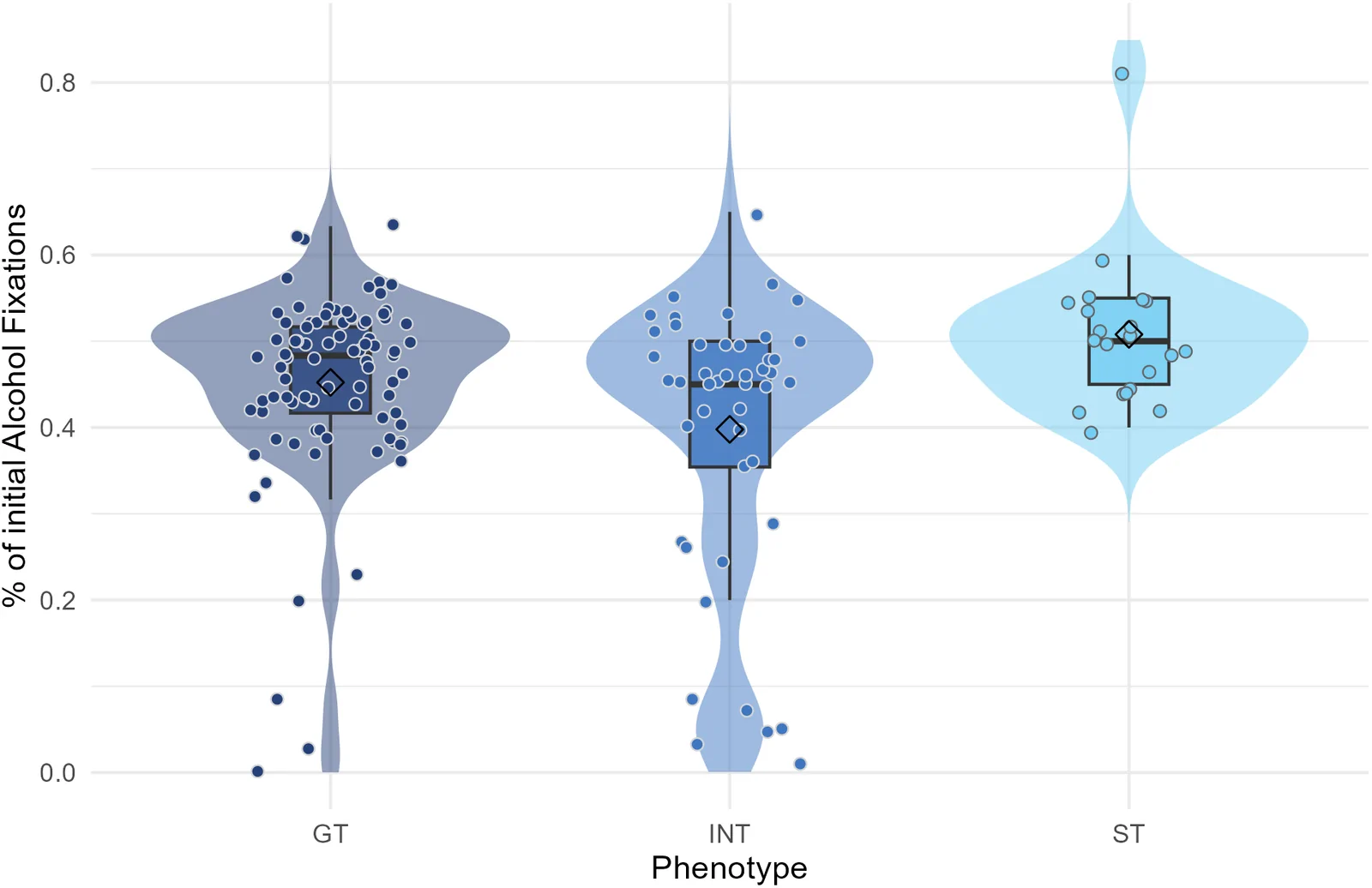
Initial orienting scores on alcohol stimuli in STs, GTs and INTs based on LPA. Horizontal lines are the medians, the lower and upper limits of the boxes are lower and upper quartiles, the ends of the whiskers represent the lower and upper extremes. The lines around the boxes represent the distribution of the data in each group.

We also examined whether the ST/GT profiles differed in *controlled attention* operationalized as the proportion of total fixation time on alcohol-related images. A Kruskal–Wallis test revealed no statistically significant differences among the three groups (GT, ST, INT), χ²(2) = 1.95, *p* = 0.37. Consistent with this finding, the estimated effect size was negligible (η² = 0.0003, 95%CI = −0.05–0.08), suggesting that sign-tracking phenotype accounted for little to no variance in sustained visual attention to alcohol stimuli.

Correlational analyses examining associations between alcohol consumption measures and alcohol-related attentional bias indices are reported in Table 4. Small-to-moderate statistically significant positive correlations were observed between all alcohol consumption–related variables and total fixation time on alcohol-related stimuli (Spearman’s ρ = .20–.25). All associations remained statistically significant following adjustment for multiple comparisons using the Benjamini-Hochberg procedure. In contrast, no significant associations were observed for rapid attentional orienting, as indexed by the percentage of first gazes directed toward alcohol-related stimuli.

**Table 4:** Correlation Coefficients, P-values and Confidence Intervals for Spearman Correlations Between Alcohol Attentional Bias Variables and Alcohol-Related Variables.

*Total Alcohol Gaze (Attentional Bias)*					
Variable	Spearman Rho	Adjusted P-Value	Raw P-Value	CI Lower	CI Upper
AUDIT	0.20	0.03*	0.01*	0.04	0.35
BATCAP (Alcohol)	0.26	0.01*	0.00*	0.10	0.41
DrinkOccas	0.18	0.05	0.03*	0.02	0.33
AUDIT-C	0.21	0.03*	0.01*	0.06	0.36
***Percent of First Alcohol Gazes***					
AUDIT	0.09	0.25	0.22	−0.06	0.25
BATCAP (Alcohol)	0.11	0.25	0.20	−0.06	0.28
DrinkOccas	0.09	0.27	0.28	−0.07	0.24
AUDIT-C	0.12	0.22	0.14	−0.04	0.27

*Note.* BATCAP (alcohol) = alcohol-related problems (assessed with the Brief Assessment Tool for Compulsivity Associated Problems), DrinkOccas = recent drinking index (number of alcoholic drinks consumed during the past two weeks x number of alcohol drinking occasions during the past two weeks), AUDIT-C = short version of the Alcohol Use Disorder Identification Test (see “Questionnaires”). *p < 0.05

### Exploratory analyses: Relationships between human sign-tracking and personality- and alcohol consumption related variables

No significant correlation was observed between the ST/GT score and personality-based variables (Table 5).

**Table 5:** Correlation Coefficients, P-values and Confidence Intervals for Spearman Correlations Between the ST/GT Score and Impulsivity, reward reactivity and psychological distress in Experiment 1.

Variable	Spearman Rho	Adjusted P-Value	Raw P-Value	CI Lower (Adjusted)	CI Upper (Adjusted)
**Psych.dist**	0.02	0.93	0.74	−0.19	0.24
**RSTPQ- RR**	0.14	0.42	0.07	−0.08	0.35
**RSTPQ- Goal**	−0.01	0.93	0.84	−0.23	0.20
**RSTPQ- BIS**	0.007	0.93	0.93	−0.21	0.23
**RSTPQ- RI**	0.09	0.78	0.28	−0.14	0.30
**RSTPQ- IM**	0.15	0.42	0.05	−0.07	0.36
**UPPSP-UR**	0.12	0.42	0.11	−0.10	0.33
**UPPSP-PU**	0.05	0.93	0.49	−0.17	0.27
**UPPSP-LPre**	0.01	0.93	0.85	−0.20	0.23
**UPPSP-LPer**	0.01	0.93	0.81	−0.20	0.24
**UPPSP-SS**	−0.05	0.93	0.55	−0.26	0.17

*Note.* CI = Confidence Interval, Psych.dist = Psychological distress (depression, anxiety and stress scale DASS-21), RST-PQ = Reinforcement Sensitivity Theory of Personality Questionnaire, subdimensions: RR = reward reactivity, Goal = goal-drive persistence, BIS = behavioral inhibition system, RI = reward interest, IM = impulsivity; UPPS-P = short version of the UPPS impulsivity scale, subdimnesions: UR = urgency, PU = positive urgency, LPre = lack of premeditation, Lper = lack of perseverance, SS = sensation seeking (see section “Data Collection” - “Questionnaires”).

No significant correlations were observed between the ST/GT score and alcohol consumption-related variables (Table 6).

**Table 6:** Correlation Coefficients, P-values and Confidence Intervals for Spearman Correlations Between the ST/GT Score and Alcohol-Consumption related Variables in Experiment 1.

Variable	Spearman Rho	Adjusted P-Value	Raw P-Value	CI Lower (Adjusted)	CI Upper (Adjusted)
**BATCAP (Alcohol)**	0.02	0.96	0.83	−0.19	0.22
**DrinkOccas**	0.00	0.96	0.96	−0.19	0.19
**AUDIT-C**	−0.06	0.96	0.44	−0.25	0.13

*Note.* BATCAP (alcohol) = alcohol-related problems (assessed with the Brief Assessment Tool for Compulsivity Associated Problems), DrinkOccas = recent drinking index (number of alcoholic drinks consumed during the past two weeks x number of alcohol drinking occasions during the past two weeks), AUDIT-C = short version of the Alcohol Use Disorder Identification Test (see section *“Questionnaires”*).

## Discussion

This study aimed to examine the potential relationship between attentional bias and sign-tracking, as well as to replicate previously observed differences in alcohol consumption across ST, GT, and INT groups [30]. An association between sign-tracking and attentional bias was theoretically expected, given that both constructs are often assumed to reflect closely related incentive-motivational processes. According to incentive salience accounts, both sign-tracking and attentional bias are thought to arise from an enhanced attribution of motivational value to reward-predictive cues, leading to increased attentional and behavioral orienting toward such stimuli [6].

Overall, the present results provide preliminary evidence for a modest relationship between sign-tracking and the initial orienting of attention toward alcohol-related stimuli. In contrast, no significant differences emerged between ST/GT profiles in alcohol consumption, and continuous ST/GT scores showed little association with alcohol- or personality-related measures. Finally, indices of maintained attention toward alcohol cues were consistently associated with several measures of alcohol use and alcohol-related problems.

### Sign-tracking and alcohol consumption

Contrary to our expectations and previous findings [30], alcohol consumption did not differ significantly between ST, GT, and INT profiles. Nevertheless, the observed pattern (FIG 2) was generally consistent with prior work, with STs displaying seemingly higher AUDIT-C scores than GTs and INTs. The corresponding effect size was small, and the relatively limited number of participants classified as STs may have reduced statistical power to detect subtle group differences. Correlational analyses using the continuous ST/GT score similarly failed to reveal robust associations with alcohol- or personality-related measures. A small positive, but statistically non‑significant, correlation with impulsivity nonetheless aligned with previous findings [26,29,34,77], but see [78].

Taken together, the present results do not provide strong support for a relationship between sign-tracking and alcohol consumption. However, neither do they constitute compelling evidence against such an association. Rather, they suggest that any relationship may be weaker and less robust than initially assumed, requiring larger and more adequately powered studies to establish its magnitude and reliability.

### Sign-tracking and alcohol-related attentional bias

The primary objective of the study was to determine whether sign-tracking tendencies are associated with attentional bias toward alcohol-related cues. Consistent with this prediction, STs exhibited a greater proportion of first gazes toward alcohol-related stimuli than INTs, indicating a modest increase in initial attentional orienting toward alcohol cues. Although the effect size was small and should therefore be interpreted cautiously, its direction is consistent with theoretical accounts of sign-tracking.

According to incentive salience theory, sign-trackers attribute enhanced motivational value to reward-predictive cues, resulting in increased attentional and behavioral attraction toward such stimuli [6]. From this perspective, alcohol drinkers with stronger sign-tracking tendencies might be expected to display greater attentional capture by alcohol-related cues. The present findings provide preliminary support for this hypothesis.

The observed association also aligns with previous suggestions that some eye-tracking based attentional bias measures (e.g., the frequency of saccades toward and away from alcohol-related stimuli) may capture processes conceptually related to sign-tracking [40].

Further support for a potential overlap between these constructs comes from studies showing that heavy drinkers often display attentional biases not only toward alcohol-related cues but also toward other appetitive stimuli more broadly [45,79]. This pattern has typically been interpreted as a generalization or “spill-over” effect, whereby repeated alcohol use enhances the motivational significance of reward-related cues in general. However, the opposite causal direction remains equally plausible. Individuals who already exhibit heightened attentional attraction to reward-predictive cues (a core characteristic of STs) may be more likely to engage in heavy drinking and subsequently develop alcohol-specific attentional biases. Longitudinal research will be required to determine the direction of these relationships.

However, the magnitude of the association observed in the present study was modest. Consequently, replication in larger and more balanced samples is needed before firm conclusions can be drawn regarding the role of sign-tracking in alcohol-related attentional bias.

### Attentional bias as a marker of alcohol involvement

While sign-tracking showed no significant relationship with alcohol-related variables, indices of maintained attention toward alcohol cues were consistently associated with alcohol consumption and alcohol-related problems. Specifically, longer dwell times on alcohol-related stimuli correlated with AUDIT scores, BATCAP scores, and recent drinking behavior. These findings are consistent with previous research indicating that attentional bias in addiction is more reliably reflected in sustained attentional engagement than in early orienting processes [37,44].

Theoretically, maintained attention may reflect the acquired motivational significance of alcohol cues resulting from repeated consumption. Repeated alcohol use is thought to sensitize motivational systems and increase the salience of alcohol-related stimuli, thereby promoting sustained attentional engagement with those cues [3,80]. Compared with rapid orienting responses, prolonged attentional engagement may provide a broader window for alcohol-related cognitions, craving-related processes, and cue-reactivity to unfold [81].

In sum, maintained attentional bias was associated with alcohol involvement, whereas sign-tracking was not. This pattern suggests that sustained attention to alcohol-related cues may function primarily as a marker of alcohol use history and acquired motivational significance rather than as a direct behavioral expression of a pre-existing vulnerability factor such as sign-tracking. Although sign-tracking and attentional bias may partially overlap conceptually, they may capture distinct aspects of addiction vulnerability and alcohol-related learning.

### Methodological considerations regarding human sign-tracking paradigms

One of the most striking observations in the present study was the distribution of latent profiles. In contrast to our previous study [30], which produced relatively balanced proportions of STs, GTs, and INTs, the present sample was predominantly classified as GTs. This discrepancy is noteworthy because both studies employed highly similar procedures.

Several explanations may account for these differences. First, latent profile classifications are inherently sample-dependent and may vary across studies even when identical analytical procedures are used [26]. Second, the relatively modest posterior probabilities observed in the present study suggest weaker separation between latent classes, raising uncertainty regarding profile membership. More fundamentally, the variability across studies may indicate that human sign-tracking and goal-tracking are better conceptualized as dimensional tendencies rather than discrete categories.

More generally, the human sign-tracking literature has produced highly inconsistent phenotype distributions [23]. Standardizing PCA paradigms and applying data‑driven clustering approaches [82] have been proposed as steps toward improving reliability. While some studies report relatively balanced groups [29,83], others find predominantly sign-tracking samples [24,25], whereas still others observe a predominance of intermediate responders [26]. These inconsistencies suggest that phenotype classification may be particularly sensitive to task design, sample characteristics, reward parameters, and analytical methods. Consequently, the true prevalence and stability of sign- and goal-tracking tendencies in humans remain uncertain.

Characteristics of the present task may also have constrained the expression of sign-tracking behavior. Unlike many animal autoshaping paradigms, the task relied on passive observation and used a relatively abstract reward structure based on virtual coins and badge collection. Although point-based rewards were effective in other paradigms [33], the absence of meaningful performance contingencies may have reduced participant engagement and limited the attribution of incentive value to predictive cues.

A related possibility concerns reward salience. In our earlier proof-of-concept study [30], participants were entered into a lottery for a €20 voucher, which may have increased motivation despite being unrelated to task performance. Because this incentive was removed in the present study following changes in ethical guidelines, participants may have perceived fewer meaningful stakes during the task. Future studies should directly examine whether reward magnitude and incentive structure influence the emergence of sign-tracking behavior in humans.

More broadly, these issues highlight the challenges of translating animal sign-tracking paradigms into human research contexts [84,85]. Unlike laboratory animals, human participants bring expectations, interpretations, and motivational concerns into experimental settings [86–88]. Consequently, how participants understand a task may substantially influence behavioral outcomes. Future studies may therefore benefit from incorporating qualitative assessments, post-task interviews, or other measures of participant engagement to better understand how human participants experience sign-tracking paradigms.

Finally, the field would benefit from developing paradigms with greater ecological validity. Human studies currently employ a wide range of rewards and conditioned stimuli, including food, money, points, toys, and abstract visual cues [23]. Yet many of these stimuli may possess limited motivational relevance in everyday life. In contrast, naturally occurring appetitive stimuli, such as alcohol cues for drinkers, may provide a more ecologically meaningful framework for assessing individual differences in attentional attraction to reward-related cues. From this perspective, established attentional bias paradigms may offer a promising complementary approach for studying sign-tracking-like processes in humans.

### Limitations and future directions

Several limitations should be acknowledged. First, the relatively small proportion of STs reduced statistical power for profile-based analyses. Second, uncertainty surrounding latent class membership may have attenuated group differences. Third, the cross-sectional design precludes conclusions regarding the causal relationship between sign-tracking, attentional bias, and alcohol use. Finally, the observed association between sign-tracking and attentional orienting was small and requires independent replication.

Future research should prioritize longitudinal designs capable of determining whether attraction to reward-predictive cues precedes alcohol use or emerges as a consequence of repeated substance exposure. Efforts to standardize human PCA paradigms and directly compare different measures of incentive salience, attentional bias, and sign-tracking would also help clarify the extent to which these constructs reflect overlapping or distinct psychological mechanisms.

## Conclusions

The present study provides preliminary evidence for a positive association between sign-tracking tendencies and the initial orienting of attention toward alcohol-related cues. Although the observed effect was small, it is consistent with theoretical accounts suggesting that individuals who attribute greater incentive salience to reward-predictive cues may also display enhanced attentional capture by substance-related stimuli [6]. As the first study to directly examine this relationship in humans, these findings represent an important initial step. Nevertheless, the small effect size warrants caution, and independent replication is required before firm conclusions can be drawn regarding the nature and strength of this association.

## Supporting information

S1 Appendix 1 Secondary study with a “manipulable” lever: methods, results and discussion. (DOCX)

S2 Appendix 2 Additional Information on Stimuli Selection for AAB task (pilot). (DOCX)

S3 Appendix 3 Gaze Evolution Analyses. (DOCX)
