# Supplementary material for: Human sign-tracking and its relationship with attentional biases for alcohol
Source: PLoS One. 2026 Sep 28;21(9):e0358192. doi: 10.1371/journal.pone.0358192 (PMC13618910; doi:10.1371/journal.pone.0358192)
Supplement: Supplementary file 1 [file pone.0358192.s001.docx]

**Supporting Information**

**S1. Appendix 1: Secondary study with a “manipulable” lever: methods, results and discussion**

**Rationale**

To increase the translational similarity of our paradigm to rodent sign-tracking procedures, we conducted a variant of the main experiment, using a movable lever cue. In animal models, sign-tracking is typically characterized by approach and interaction with a lever that predicts reward delivery. Although introducing a movable lever allowed us to more closely approximate this procedure, it remained unclear whether such a modification would elicit comparable behavior in humans. Indeed, while rodents can physically approach and engage with the lever, the lever in the present task was activated through gaze alone. Because attentional orienting and physical approach may reflect partly distinct processes, this secondary study aimed to examine whether a more animal-like cue would influence the patterns observed in the original paradigm.

Materials and Methods

## ***Participants***

The sample of this study included 138 volunteers (85 females, 50 males and 3 identified as non-binary; mean age = 23.46, sd = 3.60, see Table S1 for sample description). They completed an online screening survey to check for inclusion criteria, which were: no history of severe neurological diseases, no regular substance use (more than twice in the last two months, except for tobacco and alcohol), no colorblindness, no medicated attention deficit hyperactivity disorder. All participants had to be aged between 18 and 35 and be at least occasional alcohol consumers (abstinent people were not included). All participants had normal or corrected-to-normal vision. Overall, 180 subjects took part in the whole study. Four were excluded because they failed the attention check item, thus being considered careless responders. One was excluded due to human error. Finally, 37 subjects were excluded from the analysis due to at least one of the data quality check criteria.

**Table S1.** *Sample Description of Experiment 1*

| Variable | Median (std) | Min-Max |
| --- | --- | --- |
| Age | 23 (3.60) | 18-35 |
| Education Level | 15 (2.29) | 7-22 |
| AUDIT-C | 4 (2.30) | 0-10 |
| Recent Drinking Index | 6 (70.15) | 0-810 |

*Note.* Education Level = number of successful school years starting from elementary school; AUDIT-C= short version (consumption) of the Alcohol Use Disorder Identification Test, Recent drinking index = number of alcoholic drinks consumed during the past 2 weeks x number of drinking occasions during the past 2 weeks.

Recruitment, consent, and study title were the same as in the main study. All participants gave written informed consent to take part in the study and had the chance to participate in a price draw to win a 20-euro gift voucher. Recruitment and testing took place from February 2024 to August 2024.

## ***Procedure & Data Collection***

The procedure and materials were identical to those used in the main study, with the exception of the visual stimuli for the sign and goal.

***Goal and Sign Pictures.*** The sign (CS) was a picture of lever, which moved on the opposite direction each time the participants looked at it. The goal was a grey rectangle in which the coin rewards were delivered (see Fig S1 for a visualization of these stimuli). The sign and goal pictures were located on middle top or middle bottom of the screen and counterbalanced for location across participants.

**Figure S1.** Illustration of the sign and goal stimuli on the computer screen.

## ***Statistical Analyses***

All analyses were the same as in the main study. There were missing data for the attentional bias task due to an insufficient number of trials with gazes recorded in the ROIs (defined as at least one third of trials). Participants with missing attentional bias data were excluded from the relevant analyses (N = 7). The same indicator-reduction strategy as in the main study was applied. Specifically, highly correlated indicators were combined for the sign and middle ROIs (sign-directed fixation count and dwell time, *r* = .98; middle-zone fixation count and dwell time, *r* = .97), whereas goal-directed fixation count and dwell time were retained as separate indicators given their more modest correlation (*r* = .44). Intercorrelations among the final indicators included in the LPA are presented in Table S2.

**Table S2.**

***Intercorrelations among LPA indicators***

| **Variable** | **1** | **2** | **3** | **4** |
| --- | --- | --- | --- | --- |
| 1. Sign attention | — |  |  |  |
| 2. Middle attention | 0.17* | — |  |  |
| 3. Goal dwell time | -0.70** | -0.22* | — |  |
| 4. Goal fixation count | -0.51** | -0.11 | 0.44** | — |

*Note. *p<0.05,* ***p*<0.001.

**Results**

***Hypothesis 1. Are the “sign-tracker profiles” related to alcohol consumption levels?***

The best model based on fit indices was the 3-profile model: AIC = 1034.57, BIC = 1104.83, entropy = 0.94.

Table S3 shows the fit indices for the three models. Posterior probabilities (i.e., probabilities for each participant to belong to each of the three profiles) indicated highly mediocre confidence in the most likely profile membership for profiles 1 and 2. For profile 1, the probabilities ranged from 0.53 to 0.99 (mean = 0.95, sd = 0.10). For profile 2, they ranged from 0.53 to 0.99 (mean = 0.97, sd = 0.10). For profile 3, they ranged from 0.99 to 1.00 (mean = 0.99, sd < 0.0001). Figures S2 and S3 show means and standard errors for the indicator variables in the three profiles. Based on the examination of these graphs, groups 1, 2, and 3, were labeled respectively, “INT”, “GT”, and “ST”.

**Fig S2.** *Standardized Eye-tracking based Indicator variables in the Three LPA-Extracted Profiles.*

**Legend.** The figure shows the means and standard errors. **p<0.001, *p<0.05.

**Table S3.** *Model Fit Indices for the Three Latent Profile Analyses in Experiment 1*

| Tested Model | AIC | BIC | Entropy | Profile Sizes | Bootstrapped Likelihood Test (p-value) |
| --- | --- | --- | --- | --- | --- |
| 1 Profile | 1283.24 | 1324.22 | - | - | - |
| 2 Profiles | 1230.45 | 1286.07 | 0.90 | Profile 1: 50%  Profile 2: 50% | .01 |
| 3 Profiles | 1034.57 | 1104.83 | 0.94 | Profile 1: 33.33%  Profile 2: 49.28%  Profile 3: 17.39% | .01 |

*Note*. AIC=Akaike information criterion; BIC = Bayesian information criterion.

The Kruskal-Wallis rank-sum test, assessing the differences in alcohol consumption (measured through AUDIT-C, short version of the Alcohol Use Disorder Identification Test) in these profiles, did not show any significant effect (χ² = 0.68, *p* = 0.71, η^2^(H) = -0.009, 95% CI = [-0.07, -0.005]). Figure S3 shows the distribution of AUDIT-C scores in STs, GTs, and INTs, based on LPA-created profiles.

**Fig S3.** *Alcohol Consumption Level in STs, GTs and INTs based on LPA.*

**Legend.** Horizontal lines are the medians, the lower and upper limits of the boxes are lower and upper quartiles, the ends of the whiskers represent the lower and upper extremes. The lines around the boxes represent the distribution of the data in each group.

***Hypothesis 2. Is There a Relationship Between Human Sign-Tracking and Alcohol Attentional Bias?***

To test whether the attentional profiles differed in *automatic orienting* (operationalized as the proportion of trials in which participants initially fixed the alcohol-related image) an ANOVA was conducted. Assumptions of normality and homogeneity of variance were met. The analysis revealed no significant group differences, *F*(2,128) = 0.71, *p* = 0.49. The effect size was small (η² = 0.011), indicating that only 1% of the variance in initial alcohol fixations was explained by ST/GT profile.

To examine whether the attentional profiles differed in *controlled attention* (as measured by the proportion of total fixation time on alcohol-related images), a Kruskal-Wallis test was conducted since assumptions for ANOVA were not met. The analysis revealed no statistically significant difference between the three groups (GT, ST, INT), χ²(2) = 1.69, *p* = 0.42, η²[H] = -0.002.

Correlational analyses between alcohol consumption measures and alcohol-related attentional bias variables are shown in Table S4. Although a positive correlation was observed between AUDIT-C scores and the total fixation time on alcohol-related stimuli, this association did not remain statistically significant after applying the Benjamini-Hochberg correction for multiple comparisons.

**Table S4.** *Correlation Coefficients, P-values and Confidence Intervals for Spearman Correlations Between Alcohol Attentional Bias Variables and Alcohol-Related Variables*

| *Total Alcohol Gaze (Attentional Bias)* | | | | | |
| --- | --- | --- | --- | --- | --- |
| Variable | Spearman Rho | Adjusted P-Value | Raw P-Value | CI Lower (Adjusted) | CI Upper (Adjusted) |
| AUDIT | 0.05 | 0.579 | 0.580 | -0.19 | 0.28 |
| BATCAP (Alcohol) | 0.05 | 0.579 | 0.578 | -0.20 | 0.30 |
| DrinkOccas | 0.11 | 0.317 | 0.199 | -0.13 | 0.34 |
| AUDIT-C | 0.19 | 0.112 | 0.026* | -0.05 | 0.41 |
| Percent of First Alcohol Gazes | | | | | |
| AUDIT | -0.07 | 0.576 | 0.432 | -0.30 | 0.17 |
| BATCAP (Alcohol) | -0.19 | 0.112 | 0.042* | -0.42 | 0.06 |
| DrinkOccas | -0.18 | 0.112 | 0.041* | -0.40 | 0.06 |
| AUDIT-C | -0.14 | 0.224 | 0.112 | -0.36 | 0.10 |

*Note.* BATCAP (alcohol) = alcohol-related problems (assessed with the Brief Assessment Tool for Compulsivity Associated Problems), DrinkOccas = recent drinking index (number of alcoholic drinks consumed during the past two weeks x number of alcohol drinking occasions during the past two weeks), AUDIT-C = short version of the Alcohol Use Disorder Identification Test (see “Questionnaires”). *p<0.05, ***p<0.001

***Exploratory Analyses: Relationships between Human Sign-Tracking and Personality- and Alcohol Consumption Related Variables***

No significant correlation was observed between the ST/GT score and personality-based variables (Table S5).

**Table S5.** *Correlation Coefficients, P-values and Confidence Intervals for Spearman Correlations Between the ST/GT Score and Impulsivity, reward reactivity and psychological distress in Experiment 1*

| Variable | Spearman Rho | | Adjusted  P-Value | Raw  P-Value | | CI Lower (Adjusted) | CI Upper (Adjusted) |
| --- | --- | --- | --- | --- | --- | --- | --- |
| Psych.dist | 0.03 | | 0.876 | 0.717 | -0.21 | | 0.27 |
| RSTPQ- RR | 0.11 | | 0.876 | 0.184 | -0.13 | | 0.34 |
| RSTPQ- Goal | | 0.05 | 0.876 | 0.573 | -0.19 | | 0.29 |
| RSTPQ- BIS | 0.03 | | 0.876 | 0.705 | -0.21 | | 0.27 |
| RSTPQ- RI | -0.04 | | 0.876 | 0.625 | -0.28 | | 0.20 |
| RSTPQ- IM | 0.03 | | 0.876 | 0.699 | -0.21 | | 0.27 |
| UPPSP-UR | -0.01 | | 0.916 | 0.871 | -0.25 | | 0.22 |
| UPPSP-PU | 0.009 | | 0.916 | 0.916 | -0.23 | | 0.25 |
| UPPSP-LPre | 0.09 | | 0.876 | 0.312 | -0.16 | | 0.32 |
| UPPSP-LPer | -0.04 | | 0.876 | 0.688 | -0.27 | | 0.20 |
| UPPSP-SS | -0.06 | | 0.876 | 0.483 | -0.29 | | 0.18 |

*Note.* CI= Confidence Interval, Psych.dist = Psychological distress (depression, anxiety and stress scale DASS-21), RST-PQ = Reinforcement Sensitivity Theory of Personality Questionnaire, subdimensions: RR = reward reactivity, Goal= goal-drive persistence, BIS = behavioral inhibition system, RI = reward interest, IM = impulsivity; UPPS-P = short version of the UPPS impulsivity scale, subdimnesions: UR = urgency, PU = positive urgency, LPre = lack of premeditation, Lper = lack of perseverance, SS = sensation seeking (see section “Data Collection” - “Questionnaires”).

No significant correlations were observed between the ST/GT score and alcohol consumption-related variables (Table S6).

**Table S6.** *Correlation Coefficients, P-values and Confidence Intervals for Spearman Correlations Between the ST/GT Score and Alcohol-Consumption related Variables in Experiment 1*

| Variable | Spearman Rho | Adjusted P-Value | Raw P-Value | CI Lower (Adjusted) | CI Upper (Adjusted) |
| --- | --- | --- | --- | --- | --- |
| BATCAP (Alcohol) | 0.05 | 0.598 | 0.598 | -0.17 | 0.26 |
| DrinkOccas | 0.09 | 0.419 | 0.280 | -0.11 | 0.29 |
| AUDIT-C | 0.13 | 0.371 | 0.124 | -0.07 | 0.33 |

*Note.* BATCAP (alcohol) = alcohol-related problems (assessed with the Brief Assessment Tool for Compulsivity Associated Problems), DrinkOccas = recent drinking index (number of alcoholic drinks consumed during the past two weeks x number of alcohol drinking occasions during the past two weeks), AUDIT-C = short version of the Alcohol Use Disorder Identification Test (see section *“Questionnaires”*).

**Discussion**

From a translational perspective, we aimed to advance our PCA task further by incorporating an interactive virtual lever (similar to that used in animal studies) into the paradigm. This lever was designed to move in response to participants’ gaze, based on evidence from animal research that the type of CS (discrete and manipulable) plays a critical role in sign-tracking behavior [1,2].

In this pre-registered study, none of our hypotheses were confirmed. Unlike our previous work, where we successfully identified ST, GT, and INT groups using LPA, the posterior probabilities for group membership in the current study were suboptimal for the “GT” and “INT” profiles. Moreover, the distribution of participants across groups differed from our earlier findings (previous study: GT = 38%, ST = 33%, INT = 29%; current study: GT = 50%, ST = 19%, INT = 31%). These discrepancies alone do not invalidate the present study, as LPA-based group assignments are task-specific and reflect attentional patterns under the experimental conditions employed [3].

No significant associations were observed between sign-tracking and alcohol consumption levels, nor with personality traits theoretically linked to sign-tracking (e.g., impulsivity, reward sensitivity). Likewise, no relationship was observed between sign‑tracking and attentional bias. However, given the uncertainty surrounding the accurate identification of STs and GTs due to modifications to the PCA task, strong conclusions based on these null findings cannot be drawn. Also, the attentional bias task did not yield the expected relationships with alcohol-related variables (neither for automatic nor sustained attention) despite theoretical and empirical evidence suggesting such links [4,5]. It is further possible that the lever‑related interference observed in the PCA task carried over to the subsequent attentional‑bias task, thereby disrupting participants’ visual behavior and obscuring the expected patterns as a function of their alcohol‑use levels.

We hypothesize that the modifications implemented in this study may have significantly changed participants’ strategies and overall engagement with the task. Specifically, the use of a gaze-controlled symbolic lever on a screen differs substantially from the physical, manipulable lever commonly employed in rodent studies. This discrepancy may partly explain the null findings observed here. Also, the interactive lever may have prompted an operant-like approach, leading participants to believe that rewards were contingent on moving the lever. This interpretation is based on learned human experiences, where manipulating a lever typically produces an outcome (analogous to flipping a switch), unlike naïve rodents for which the lever is initially a neutral object prior to conditioning. In this case, the sign would no longer function as a simple CS signaling the reward, but rather as a manipulandum within an operant framework. Under such conditions, it would no longer be possible to distinguish STs from GTs on the basis of the incentive salience attributed to a CS. To clarify this issue, future studies should include a cognitive interviewing phase at the end of the experiment to probe participants’ perceived contingencies, the role they attributed to the lever, and their strategies during the task. Such qualitative data, unavailable in animal studies, could provide valuable insights, while acknowledging that unconscious processes may exert effects distinct from participants’ explicit beliefs.

Translating animal behavioral paradigms into human research remains challenging [6,7]. Beyond practical constraints such as constructing manipulable apparatuses, human participants bring interpretive processes into the experiments [8–10]. For example, operating a lever carries pre‑existing meaning for humans (typically associated with producing a consequence), and social norms (e.g., do not touch unfamiliar equipment) might reduce interaction with physical experimental devices.

**References**

1.	Meyer PJ, Cogan ES, Robinson TE. The Form of a Conditioned Stimulus Can Influence the Degree to Which It Acquires Incentive Motivational Properties. PLOS ONE. 2014;9: e98163. doi:10.1371/journal.pone.0098163

2.	Roughley S, Killcross S. Differential involvement of dopamine receptor subtypes in the acquisition of Pavlovian sign-tracking and goal-tracking responses. Psychopharmacology (Berl). 2019;236: 1853–1862. doi:10.1007/s00213-019-5169-8

3.	Cope L, Gheidi A, Martz ME, Duval ER, Khalil H, Allerton T, et al. A mechanical task for measuring sign- and goal-tracking in humans: A proof-of-concept study. Behav Brain Res. 2023;436: 114112. doi:10.1016/j.bbr.2022.114112

4.	Bollen Z, Field M, Billaux P, Maurage P. Attentional bias in alcohol drinkers: A systematic review of its link with consumption variables. Neurosci Biobehav Rev. 2022;139: 104703. doi:10.1016/j.neubiorev.2022.104703

5.	Field M, Cox W. Attentional bias in addictive behaviors: A review of its development, causes, and consequences. Drug Alcohol Depend. 2008;97: 1–20. doi:10.1016/j.drugalcdep.2008.03.030

6.	Crombag HS, Duka T, Stephens DN. The Continuing Challenges of Studying Parallel Behaviours in Humans and Animal Models. In: Sommer WH, Spanagel R, editors. Behavioral Neuroscience of Alcohol Addiction: Translational Studies and Human Phenotypes. Cham: Springer Nature Switzerland; 2025. pp. 551–594. doi:10.1007/7854_2024_485

7.	Stephens DN, Crombag HS, Duka T. The Challenge of Studying Parallel Behaviors in Humans and Animal Models. In: Sommer WH, Spanagel R, editors. Behavioral Neurobiology of Alcohol Addiction. Berlin, Heidelberg: Springer; 2013. pp. 611–645. doi:10.1007/978-3-642-28720-6_133

8.	Jimenez-Buedo M, Guala F. Artificiality, Reactivity, and Demand Effects in Experimental Economics. Philos Soc Sci. 2016;46: 3–23. doi:10.1177/0048393115618015

9.	Nichols AL, Maner JK. The Good-Subject Effect: Investigating Participant Demand Characteristics. J Gen Psychol. 2008;135: 151–166. doi:10.3200/GENP.135.2.151-166

10.	Orne MT. On the social psychology of the psychological experiment: With particular reference to demand characteristics and their implications. Am Psychol. 1962;17: 776–783. doi:10.1037/h0043424
