# Supplementary material for: Human sign-tracking and its relationship with attentional biases for alcohol
Source: PLoS One. 2026 Sep 28;21(9):e0358192. doi: 10.1371/journal.pone.0358192 (PMC13618910; doi:10.1371/journal.pone.0358192)
Supplement: Supplementary file 2 [file pone.0358192.s002.docx]

**S2.** **Appendix 2: Additional Information on Stimuli Selection for alcohol attentional bias task (pilot).**

| Category | Mean score for Neutral Pictures (range) | Mean score for Alcohol Pictures (range) |
| --- | --- | --- |
| Beer (N=13) | 0.82 (0.23-3.85) | 9.20 (8.31-9.46) |
| Champagne (N=13) | 0.55 (0.23-1.31) | 9.38 (9.31-9.46) |
| Cocktail (N=13) | 0.81 (0.23-2.15) | 9.05 (8.77-9.62) |
| Environment (N=13) | 0.88 (0.23-2.62) | 9.02 (8.23-9.62) |
| Liquor (N=13) | 0.89 (0.31-1.85) | 9.46 (9.15-9.85) |
| Wine (N=16) | 0.92 (0.18-3.43) | 8.51 (7.69-9.06) |

**Table S7**.

*Mean Alcohol-Relatedness Scores by Stimulus Category (Pilot phase).*
