# Supplementary material for: Human sign-tracking and its relationship with attentional biases for alcohol
Source: PLoS One. 2026 Sep 28;21(9):e0358192. doi: 10.1371/journal.pone.0358192 (PMC13618910; doi:10.1371/journal.pone.0358192)
Supplement: Supplementary file 3 [file pone.0358192.s003.docx]

**S3. Appendix 3: Gaze Evolution Analyses**

To examine whether dwell time (toward the sign and the goal) changed across trials and experimental blocks for the three behavioral profiles (extracted via LPA), we fitted a linear mixed‑effects model including fixed effects of profile, trial number, block, and their interactions (profile × trial and profile × bloc). The model included a random intercept and a random slope of trial number for each participant to account for individual differences in overall dwell time and learning trajectories. To visualize how the profiles evolved in dwell time through the task, we plotted their dwell time on the first vs the last trial (Figures S4 and S5).

Regarding **dwell time toward the goal**, all main effects and interactions were highly significant (all *p* < .001), with the exception of the effect of *bloc* (*p* = .12). Visual inspection indicated that GTs exhibited high levels of goal-directed responding throughout both blocks, with a slight increase from the first to the last trial. In contrast, STs consistently displayed minimal goal-directed responding across trials. INTs showed greater heterogeneity, with substantial between-subject variability and both increases and decreases over time. In both Blocks 1 and 2, all pairwise comparisons between profiles were significant (all *p* < .0001).

Regarding **dwell time toward the sign**, all main effects and interactions were significant (all *p* < .05), with the exception of the effect of *trial* (*p* = .07). Examining the change from the first to the last trial in both blocks shows that STs maintained high levels of sign-directed responding and showed a modest increase from the first to the last trial. Conversely, GTs exhibited a decline in sign-directed responding, with scores clustering near zero by the final trial. INTs displayed intermediate but variable levels of responding, with Block 2 characterized by predominantly low sign-directed scores and a small number of higher-responding individuals. In both Blocks 1 and 2, all pairwise comparisons between profiles were significant (*p* < .05), except for the comparison between GTs and INTs in Block 2 (*p* = .05).

**Figure S4. Difference in Goal Dwell Time between First and Last Trials, in Blocs 1 and 2**

**Figure S5. Difference in Sign Dwell Time between First and Last Trials, in Blocs 1 and 2**
